# Use of Social Media By Nurse Educator Students: An Exploratory Survey

**DOI:** 10.2174/1874434601711010026

**Published:** 2017-02-28

**Authors:** Mari Lahti, Heidi Haapaniemi-Kahala, Leena Salminen

**Affiliations:** 1 Post-Doc researcher, Department of Nursing Science, University of Turku, Finland, Senior lecture in Turku University of Applied Science, Finland; 2 Department of Nursing Science, University of Turku, Finland; 3 University Lecturer, Department of Nursing Science, University of Turku, Finland

**Keywords:** Nurse educator student, Social media, Social media needs, Nurse teacher education, Education, SPSS

## Abstract

**Background:**

Information and Communication Technology (ICT) opens up new possibilities for introducing innovative teaching and learning methods to deliver education in different educational areas. Use of internet and social media has grown rapidly and is a key way in how nurses and nurse educator students find information. However, the evidence is still lacking how nurse educator students use social media.

**Objective:**

The aim of this study is to describe nurse educator students’ use of social media and the ways in which their educational needs are related to social media.

**Method:**

The data were collected using a structured questionnaire that included one open question. Altogether, 49 nurse educator students completed and returned the questionnaire (response rate 96%). The quantitative data were analysed using statistical programme SPPS and content analysis.

**Results:**

While many nurse educator students reported using the tools of social media, others claimed that they do not use social media at all. Facebook was most common (53% use it every day) and YouTube (17%) the second most common form of social media used to support daily living. The participants reported using YouTube (6% use it every day) and Facebook (4%) most often as support in their studies. They reported using Second life as virtual reality form of social media, the least. The most common educational needs of nurse educator students include receiving more in-depth information about how to use social media, as well as more practice in using it.

**Conclusion:**

In the future, the education of the nurse educator students should include even more in-depth information about the forms of social media and about the advantages of using it in teaching. The education should encourage nurse educator students and provide them with more possibilities to train and make use of the benefits of social media as support in their daily lives and studies. There is need for more robust evidence of social media use in nurse educator students education.

## INTRODUCTION

Information and Communication Technology (ICT) opens up new possibilities for introducing innovative teaching and learning methods to deliver education in different educational areas [1]. It is being increasingly used in the field of education and provides a new way of delivering education in general [1-3]. New and advanced teaching methods have also been introduced in the field of health care and nursing of late. Nursing and also other health care students in fact tend to use the new forms of social media many hours a day [4, 5]. Social media was understood as a wider term including collaborative projects (*e.g.* user generated content, content communities, content sharing and social online networking sites), social networking meaning as Facebook, Twitter, YouTube, Instagram, blog, Wiki and chat. [6] Therefore, health care educators need to update their traditional pedagogical methods so that they conform more to the current era of technology [5, 7].

Internet use is fairly common throughout Europe: in 2014, about 71% of Europeans reported that they use the Internet, whereas in the same year about 95% of Finnish adults said that they had used the Internet during the past three month period [8]. Two-thirds of adult Internet users report using social media websites. This number has increased dramatically over the last few years. [8] The Internet is a key way in which health care nurses find information: 83% claim that they use the Internet to find information. But even still, the use of social media among nurses lags behind the average for all adults. Only 11% of nurses report using social media. [9] However, health care educators’ use of social media in teaching is still limited [5, 10]. For example, social media can be used as educational means by having reflective journal blogs or having closed groups on Facebook to share learning experiences [12]. Moreover, research related to use of social media in nurse educator students teaching and learning is lacking.

There are several new and advanced ways of using ICT and creating alternative teaching methods [13], which can be applied to nursing and health care education [14, 15]. Social media is one of these new ways of teaching. Social media is defined by three elements: content, groups and use of the Internet. Content can include text, pictures or videos published on Internet. Groups are the second important element in social media; they offer ways in which places where people can communicate with each other. The last element is the Internet itself: without the Internet there would be no social media. [16] Social media can also be defined by websites that allow individuals to have a public profile on an Internet website and communicate with other users that share a common website [17].

Social media tools such as YouTube, Facebook, blogs, wikis and Second Life are interactive communication tools that are easy to use and can be used in learning [13, 18]. YouTube is a form of social media that contains personal TV, video and music clips, which anyone can upload [7]. Facebook is one form of social media, and it allows users to download videos, text and to communicate with one another. There are more than 120 million Facebook users daily. Clifton and Mann [7] have described the benefits of using YouTube in nursing education, arguing that YouTube allows nursing students to combine, compare and analyze their own ideas. It also brings out several different points of view, stimulates discussion and helps train both knowledge and critical thinking abilities [7, 19]. Also, Billings [18] argues that wikis and blogs have several good aspects to them, such as facilitating dialog and the communication between nursing students and educators. In addition, students can develop their team working skills and learn how to maximise the possibilities of teamwork with the help of the network communication offered by wikis [20]. In the Second Life, the student can be trained, for example, in decision-making [21]. Second life is part of Virtual reality (VR) which is a modern, experimental, computerised and real-time technology that uses visual graphics, sounds and other sensory input which creates an interactive computer world. [22]

The challenge for the future in higher education is that more focus should be placed on the educator’s role and on the use of social media [23]. Social media presents new challenges for educators as today’s students are increasingly using these new systems and educators should be prepared to work with students who have an information-age mindset. For example, if educators are not aware of these modern forms of social media, it is difficult to understand and teach with using these modern teaching methods. Emphasis should be put on educator’s level of engagement with social media use and social media should be a part of educator education qualifications. [11, 23] Furthermore, studies have reported that just as students are using different social media forms, educators are also using them in their personal life so they should be familiar to these methods and therefore to be able to adopt these to teaching routines as well. But even still, social media is rarely used in higher education teaching [5, 11].

There are also some concerning factors related to the use of social media in teaching. The existing social media tools were not originally developed for teaching purposes and especially for nursing education. [24.] Some disadvantages related to use of social media have also been reported. These disadvantages include rapid changes in the format of social media and a lack of information on the effort needed so they could be used effectively in teaching and learning. [23] According to Nokelainen [25], the pedagogical usability of digital learning materials needs to be assessed, which also touches upon the use of social media tools in general. Nokelainen [25] has described in his study one quite useful evaluation instrument which can be used as a quality measurement tool of social media or other digital learning material. There is also a lack of research on use of social media in teaching [26], and particularly in the field of health care educators’ education. To overcome the research gap, this study focuses on health care educator students” use of and educational needs regarding social media.

The aim of this study was to focus on nurse educator students’ use of and educational needs regarding social media. The research questions were as follows: 1) How often do health care educator candidates use social media (*i.e.* blogs, wikis, Second Life, YouTube, Facebook) as support in their daily lives and studies? 2) What kinds of educational needs do the health care educator candidates have with respect to social media? The ultimate goal was to obtain information about the ways in which health care educator candidates use social media and, therefore, to develop the education for health care educators in relation to social media. To the best of authors’ knowledge, this is the first study done exploring the nurse educator students’ use of social media and needs related to it. This information can be used to implement new learning and teaching approaches and methodologies while developing nurse educator students’ curricula’s further.

In Finland, a nurse educator must have a master’ s degree, three years of work experience in the health care system and 60 ECTS of pedagogic studies [27]. In this study, the term ‘nurse educator student’ refers to a university student pursuing a master’s degree with nursing science as a major subject, and health care (consists also nurse) education pedagogic studies, including the science of education, as a minor subject. They are qualified nurses or other health care professionals holding a college or polytechnic degree who are training to become educators of health care or nursing education.

## MATERIALS AND METHODS

### Design and Instrument

A descriptive, cross-sectional exploratory survey design was used [28]. The data were collected using a structured questionnaire, which was developed for this study and which is based on studies done in prior publications. The ‘Health care educator students’ use of and educational needs regarding social media’ questionnaire (EDNESOME) consisted of three different parts: 1) socio-demographic variables (4 items: age, gender, level of education and previous education); 2) use of social media as support in their daily lives and for their studies (10 items); and 3) social media related educational needs (1 item: open question). A 5-point Likert scale was used to evaluate the use of social media in daily life and studies (1=use every day, 2= use every week, 3= use several times a month, 4= use seldom than once a month, and 5= do not use at all). In this study, the following social media forms were selected: blogs, wikis, Second Life, YouTube and Facebook. They were selected because they are the most popular forms of social media in Finland. The questionnaire was pilot tested with three health care educator students’ before the data collection phase. Some items on the questionnaire were revised after the pilot study by clarifying meanings.

### Sample, Setting and Data Collection

All of the health care educator students’ selected for this study were from a single Finnish university (N=51). The education lasts for three academic years to all students’. The data were collected at the end of the year 2010 separately for all three groups, which were at different levels in their education. The data were collected at the beginning of the lectures. Information about the study was given in oral and also in written form along with a cover letter before participants began filling in the questionnaire. Altogether, 49 nurse educator students’ returned the questionnaire anonymously. The response rate was 96%.

### Data Analysis

The data were analysed using the SPSS (Statistical Package for Social Sciences) programme (version 18.0) (SPSS Inc., Chicago, IL). First, the means, standard deviations, frequencies and percentages of the descriptive statistics were used to describe the participants` socio-demographic variables and the health care educator candidates’ use of social media as support in their daily lives and studies. Second, Spearman’s correlation was used to examine the correlations between the scale questions and background variables assess social media as support in the participants’ daily lives and studies and the socio-demographic variables. In this study, p-values below 0.05 were interpreted as statistically significant [28]; only statistically significant values are reported.

The answers to the open question describing participants’ educational needs were assessed using content analysis [29]. The content analysis method used is described as follows. The units of analysis were single words, word pairs or sentences. First, the answers were recorded using Microsoft Word (2010). Second, the researcher became acquainted with the data by reading it. Third, the text was divided into meaning units and the meaning units were abstracted and labelled with a code. Fourth, the researcher sought the differences and similarities between the codes and sorted them into different subcategories. Finally, the researcher grouped the similar subcategories, named the main categories and quantified the results.

### Ethical Considerations

The basic principles of research ethics were followed at every stage of the study [30, 31]. The permission to conduct the study was approved by the head of the department of the university. Participants received oral and written information about the purpose of the study and their rights. Participation in the study was voluntary and refusal did not affect the participants’ education in any way. Completing the questionnaire was regarded as informed consent to participate in the study. The data were treated in confidence and participants’ anonymity was ensured by encrypting the data during the analysis.

## RESULTS

### Participants

A total of 49 health care educator candidates participated in this study. More than 90% of the candidates were women. The mean age of the health care educator candidates was 36 years (SD 1.7 years). Twelve of the candidate had a double degree as, for example, a midwife and a nurse or a public health nurse and a general nurse. However, these particular job descriptions were registered as something other (as the public health nurses or as the midwives or as paramedics) than nurse, so it can be noted that 39 (80%) of the health care educator candidates´ were in fact nurses Table (**1**).

### Use of Social Media as Support in Daily Living and Studies

The health care educator students’ reported using social media in their daily lives and studies quite often. Facebook was the most common form of social media used as support in their daily lives: 26 (53%) candidates reported that they use it every day and seven students (14%) said they use it weekly. The candidates reported using Facebook less often to support their studies than they did as support in their daily lives Table (**2**).

Candidates also reported using YouTube quite often as support in their daily lives, although 19% of health care educator candidates (n=9) reported that they do not use it at all. While YouTube was reportedly the most often used form of social media as support in candidates’ studies, fifteen candidates (32%) reported that they do not use it at all. Facebook was the second most common form of social media used in support of studies: 32% of the candidates (n=15) reported using Facebook every week or several times a month as support in their studies (Table **2**).

Approximately 20% of the health care educator candidates (n=9) reported using blogs every week as support in their studies. However, more than 60% of the candidates (n=29) reported that they do not use blogs at all as support in their daily lives. Candidates reported that they rarely use Second Life in their daily lives or studies. Almost all educator candidates (90%) reported that they do not use it at all (Table **2**).

The health care educator candidates’ age, gender, previous education or level of education was not statistically significant in terms of how often candidates use social media as support in their daily lives or studies (p>0.05).

### Educational Needs Related to Social Media

The educational needs of health care educator candidates can be divided in three main categories: 1) competence at using social media in teaching, 2) practical examples of the use of social media and 3) practical training in the use of social media. Slightly more than half of the health care educator candidates (n=29, 60%) reported on their educational needs concerning the use of social media. Nearly a third of the candidates (n=15) brought up the fact that they are in need of versatile and more in-depth information about the different types of social media and about how to use them in teaching. The health care educator candidates wrote that they need in-depth information about wikis, blogs, Second Life, educational videos and Facebook. Also, the need for more information about copyrights, media education and arranging network courses was brought up in the responses (Table **3**).

The need for the practical examples of how to use social media was very clearly emphasised: every fifth candidate (n=10) brought up the fact that they would need more concrete examples of how to use social media in teaching and how the different methods can be utilised and evaluated. Furthermore, candidates hoped for tips and examples of how social media has been used in various teaching situations and teaching subjects. Participants also mentioned that there is a need to assess the pedagogical usability of social media as a teaching and learning method (Nokelainen, 2006). A little more than 10% of the health care educator candidates (n=6) brought up the fact that they need practical training in how to use social media (Table **3**).

## DISCUSSION

The aim of this study was to focus on nurse educator students’ use of and educational needs regarding social media. The nurse educator students reported using social media as support in their daily lives and studies, which reflects the fact that they currently make use of the many possibilities of social media. However, quite a few nurse educator student reported that they do not use certain social media tools at all. This can be due to the fact that those particular students’ were not equipped to use them or did not have enough knowledge about the advantages of social media. When taking into account the growing popularity of virtual worlds, the fact that Second Life proved to be least popular form of social media was surprising. One of the reasons may be that nurse educator students’ are not ready or do not have enough courage to use it. Also, Second Life is quite complicated to use and requires a time to learn to use it in *e.g.* teaching classes. The health care educator candidates should be encouraged to use Second Life more because, for example, it is one of the methods used to train people in decision-making [21]. Also it can be used as a learning environment in distance learning in nurse educator education.

Educators need social media skills and, therefore, educator education could include more media education and teaching related to the different types of social media. In the future, nurse educator education should offer even more in-depth information about the different types of social media, about the advantages of such media and how to use them in teaching. Moreover, educators need to find ways of getting inside the student’s head to understand better how students use and think about these tools and clearly more research is needed here [5, 32]. In addition, educator education should offer more training in the various types of social media and, in this way, encourage candidates to use social media as support in their daily lives and studies. For example, nurse educators should use YouTube more often in education because it increases nurse students´ knowledge and critical thinking skills [7]. In addition, educator’need also to consider the educational theories that are relevant for the digital world. It is also important to include them in curriculum development and course design. [32]

The education for nurse educator students can be arranged so that it includes more social media and instructions how to use it. A learning diary in blog or wiki form, for example, could be one useful learning method in place of written seminar papers or examinations. Nurse educator students could practice their team-working skills with the help of wikis. According to previous studies [18, 20], blogs and wikis facilitate dialog and communication between students and educators [18], and wikis also develop students’ team-working skills [20]. One alternative to increase the presence of social media in education is to arrange optional courses that could be completed with the help of different types of social media, in which case the student would get more experience and training in how to use social media. In the future, it will also be important to develop nurse educators’ teaching skills and traditional pedagogical methods to better meet the new technology demands.

The use of social media as support in health care educator candidates’ daily lives and studies is still a fairly unexplored area. In general, social media has only been studied quite marginally; for example, there is a lack of research on how to use social media in teaching [26]. Further studies are needed. There is also a need for future research among health care educators on how they use social media in teaching and how competent they are in using social media in teaching. Moreover, future studies might address the use of randomised, controlled trials to test the effectiveness of these new alternative teaching methods.

### Limitations

There were some limitations in this study. First, the questionnaire was developed for this study, but in such a way that it might affect the reliability of the results [28]. However, the questionnaire was feasible and should be tested and validated in the future with larger sample sizes. This study can be seen as a preliminary study that calls for further studies. Second, the sample size was small and collected from one university and this may affect to the generalization of the results [28].Third, in this study only a few forms of social media were studied, even though they were the most used social media tools.

#### Recommendations For Nurse Educator Education

Based on the results, we make the following recommendations:

- Health care educator education should include more social media in teaching
- Health care educator education should include more possibilities to train, utilise and assess the benefits of social media as support for candidates in their daily lives and studies

## CONCLUSION

This exploratory survey on nurse educator students’ daily use of social media and educational needs for social media was supported by previous studies on the field. Similarities were found that nurse educator students’ use social media on daily basis however some findings were novel and surprising *e.g.* that Second Life is not used that much even though there are need for use of new and innovative methods in teaching and learning. This study also emphasises the need for future education to be lifted in to next generation level. There is clear need to add the social media education and training into nurse educator education in near future. Moreover, there is need for future research on the field to show more preciously in what ways social media is used in nurse educator students education and how it could be transferred into their working life.

## Figures and Tables

**Table 1:** The background factors of the respondents (N=49).

**Variable**	**n**	**%**
*Age in years* ≤ 25 26-35 36-45 ≥ 46	2 23 15 9	4 46 31 19
*Level of nurse educator education* 1. academic year 2. academic year 3. academic year other	14 16 18 1	29 32 37 2
*Previous education* Nurse Public health nurse other: (*e.g.* radiographer, midwife, paramedic, laboratory nurse, physiotherapist)	27 14 8	55 29 16

**Table 2:** Health care educator students’ use of social media as support in their daily lives and studies.

**The use of social media form**	scale	in daily living n	in daily living %	in studies n	in studies %
Blogs n=45-46
	Use every day	1	2	0	0
	Use every week	4	9	9	20
	Use several times in a month	5	11	10	21
	Use less than once a month	6	13	7	15
	Do not use at all	29	64	20	43
Wiki n=47-48	Use every day	0	0	1	2
	Use every week	7	15	7	14
	Use several times in a month	5	11	6	12
	Use less than once a month	8	17	16	34
	Do not use at all	27	57	18	38
Facebook n=46-49	Use every day	26	53	2	4
	Use every week	7	14	8	17
	Use several times in a month	0	0	7	15
	Use less than once a month	1	2	3	7
	Do not use at all	15	31	26	57
YouTube n=47-49	Use every day	5	17	3	6
	Use every week	11	23	3	6
	Use several times in a month	13	27	10	21
	Use less than once a month	10	21	15	32
	Do not use at all	9	19	15	32
Second Llife n=43-44	Use every day	1	2	1	2
	Use every week	0	0	1	2
	Use several times in a month	0	0	0	0
	Use less than once a month	3	7	8	19
	Do not use at all	40	91	33	77

**Table 3:** Health care educator students´ educational needs as they relate to social media (n=29).

Educational needs related to social media	**n**
Competence to use social media in teaching	15
Practical examples of the use of social media	10
Practical training in the use of social media	6
